# The associations of maternal and children’s gut microbiota with the development of atopic dermatitis for children aged 2 years

**DOI:** 10.3389/fimmu.2022.1038876

**Published:** 2022-11-17

**Authors:** Xiaoxiao Fan, Tianzi Zang, Jiamiao Dai, Ni Wu, Chloe Hope, Jinbing Bai, Yanqun Liu

**Affiliations:** ^1^ Wuhan University School of Nursing, Wuhan University, Wuhan, China; ^2^ Emory University Nell Hodgson Woodruff School of Nursing, Atlanta, GA, United States

**Keywords:** gut microbiota, atopic dermatitis, eczema, pregnancy, offspring

## Abstract

**Background:**

It is critical to investigate the underlying pathophysiological mechanisms in the development of atopic dermatitis. The microbiota hypothesis suggested that the development of allergic diseases may be attributed to the gut microbiota of mother-offspring pairs. The purpose of this study was to investigate the relationship among maternal-offspring gut microbiota and the subsequent development of atopic dermatitis in infants and toddlers at 2 years old.

**Methods:**

A total of 36 maternal-offspring pairs were enrolled and followed up to 2 years postpartum in central China. Demographic information and stool samples were collected perinatally from pregnant mothers and again postpartum from their respective offspring at the following time intervals: time of birth, 6 months, 1 year and 2 years. Stool samples were sequenced with the 16S Illumina MiSeq platform. Logistic regression analysis was used to explore the differences in gut microbiota between the atopic dermatitis group and control group.

**Results:**

Our results showed that mothers of infants and toddlers with atopic dermatitis had higher abundance of *Candidatus_Stoquefichus* and *Pseudomonas* in pregnancy and that infants and toddlers with atopic dermatitis had higher abundance of *Eubacterium_xylanophilum_group* at birth, *Ruminococcus_gauvreauii_group* at 1 year and *UCG-002* at 2 years, and lower abundance of *Gemella* and *Veillonella* at 2 years. Additionally, the results demonstrated a lower abundance of *Prevotella* in mothers of infants and toddlers with atopic dermatitis compared to mothers of the control group, although no statistical difference was found in the subsequent analysis.

**Conclusion:**

The results of this study support that gut microbiota status among mother-offspring pairs appears to be associated with the pathophysiological development of pediatric atopic dermatitis.

## Introduction

There has been an increased incidence in allergic diseases, including allergic asthma, allergic rhinitis, and food allergies in the last 40 years (1). Atopic dermatitis is defined as chronic inflammatory disease of the integumentary system characterized by recurring symptoms, including pruritus, dryness, peeling, blistering, bleeding, and risk for secondary bacterial infection (2). Epidemiological survey has reported 60% of atopic dermatitis occurs in the first year of life (infantile eczema) (3). Previous study (4) indicated that the prevalence of atopic dermatitis in children was as high as 30%. In China, the prevalence of atopic dermatitis in infants was estimated as 64.8% (5). Furthermore, the literature indicated that atopic dermatitis may present as an initial manifestation of an underlying allergic disease process in infants and toddlers. Of note, up to 80% of infants and toddlers diagnosed with atopic dermatitis eventually develop allergic rhinitis or asthma later in childhood (6). Therefore, it is imperative to identify factors that may influence the pathophysiological development of pediatric atopic dermatitis.

Epidemiological studies have identified a number of environmental risk factors that may play a role in the development of atopic dermatitis, such as mode of delivery, breastfeeding status, urban living factors, pet exposure, tobacco exposure, antibiotic therapy and dietary habits (7). Recent research surrounding the “microbiota hypothesis” has acquired increased attention suggesting that allergic processes may stem from an underlying imbalance within the gut microbiota (8). Prior studies indicated that the composition of the gut microbiota was an important factor in the normal development of immune system functioning (9) and that certain gut microbiota and microbial metabolites may promote the production of regulatory peripheral T cells, providing a protective effect against inflammatory processes that drive allergic and autoimmune disease processes (10–12). Moreover, these studies indicate that gut microbiota is an important regulator in the pathogenesis of atopic dermatitis (13–15).

Low diversity of gut microbiota and alternative gut microbial composition were associated with the development of atopic dermatitis in infants and toddlers compared to healthy children (16). Children with atopic dermatitis exhibit a higher abundance of *Bacillariophyceae, Clostridium* and *Enterobacteriaceae*, and a lower relative abundance of *Bifidobacterium and Lactobacillus* (16). Wang et al. (15) found that a decrease in gut microbiota diversity at one week of age was strongly associated with developing atopic dermatitis within the first 18 months of life postpartum. Recently, studies (17–19) also revealed that the maternal gut microbiota during pregnancy was crucial in the development of healthy infantile immune system functioning. Lange et al. (20) reported that higher counts of maternal total *aerobes* and *Enterococci* were associated with an increased risk for asthma-like symptoms among infants. Comprehensive analysis of both the maternal and offspring’s gut microbiota may provide insight into establishing key biomarkers for the prediction of subsequent pediatric allergic disease states, including atopic dermatitis, within the first two years of life postpartum. Interpretation of these results may provide a fulcrum on which the development of target-specific pharmaceutical therapies and interventions hinge upon.

However, some inconsistencies were noted regarding the role of gut microbiota in the development of atopic dermatitis in infants and toddlers, which may be explained by time discrepancies in stool sample collection, variable microbiological profiling methods, and poor control of potential confounding variables that may indirectly affect gut microbiota composition (8, 16). Currently, many studies exploring the relationship between gut microbiota and atopic dermatitis are cross-sectional studies with a tendency toward reverse causality, including changes in microbiota composition due to disease manifestations. Few studies have included longitudinal cohort studies linking infant microbiota status to the subsequent development of pediatric atopic dermatitis (21). Additionally, previous literature on the gut microbiota and allergic diseases have solely focused on the postnatal period, but recent findings suggest that maternal gut microbiota status during pregnancy plays a pivotal role in fetal immune development (22, 23) and may drive the evolution of allergic diseases in respective offspring (20, 24).

There remain few relevant population studies to investigate the important relationship between maternal gut microbiota during pregnancy and the risk for infantile atopic dermatitis. Therefore, this study was a 2-year prospective cohort study to investigate the relationships between maternal-offspring’s gut microbiota during pregnancy and the subsequent risk for development of atopic dermatitis in offspring up to two years postpartum.

## Materials and methods

### Study design and participants

This was a prospective cohort study. Sixty-two pregnant women were recruited from March 2017 to November 2017 in Central China and followed until two years of age. The inclusion criteria were (1): pregnant women in the third trimester, (2) pregnant women who planned to give birth in a tertiary hospital in central China, and (3) pregnant women without pregnancy complications. The exclusion criteria were: (1) pregnant women receiving antibiotic treatment, and (2) those with cognitive impairment. A total of 62 pregnant women were recruited in our study, and 21 mother-offspring pairs could not be reached by phone or email at follow-up, leaving 41 mother-offspring pairs in this cohort. Of these 41 mother-offspring pairs, one infant had asthma and four participants of sample were not collected at 6 months, 1 year, or 2 years, therefore, 36 participants were included in the final analysis of this study. This study was approved by the Research Ethics Boards of Medical School of Wuhan University (JKHL2017-03-03). Informed consent was obtained for all participants.

### Variables and measures

At the time of recruitment, pregnant women in the third trimester completed a demographic questionnaire and diet questionnaire. Newborn general demographic data, including gender, height, weight, and mode of delivery, were obtained *via* hospital medical records at the time of birth. Moreover, data collection was performed regarding pertinent perinatal and postnatal environmental exposures, including alcohol exposure, pet dander exposure, feeding modalities, and use of antibiotics up to postpartum two years.

Stool samples were collected perinatally from pregnant women in the third trimester and again postpartum from their respective offspring at the following time intervals: time of birth, 6 months, 1 year, and 2 years. Sample collection was performed by trained personnel at the participant’s home. Samples obtained consisted of formed stool or residual fecal matter collected from infant diapers in accordance to the Human Microbiota Project (HMP) protocol. Following immediate collection, all stool samples were transported to the laboratory in an incubator (+4 °C) and then were stored at -80 °C in a freezer at our laboratory. DNA extraction, polymerase chain reaction (PCR) amplification, and Illumina MiSeq sequencing about stool samples have been elaborated on our previous study (25). The V3-V4 highly variable region of the bacterial 16S rRNA gene was amplified with primers 338F (5’-ACTCCTACGGGAGGCAGCAG-3’) and 806R (5’ - ggactachvgggtwtctaat -3’) by a thermocycling PCR system (GeneAmp 9700, ABI, Walthma, MA, USA).

In this study, pediatrician used the Williams’ criteria (26) for the diagnosis of atopic dermatitis in infants and toddlers between the ages of 1 and 2 years. The Williams’ criteria were as follows: primary criteria: pruritus; secondary criteria: (1) history of flexor side dermatitis eczema, including elbow fossa, rouge fossa, anterior ankle, and neck (children under 10 years old including buccal rash); (2) history of asthma or allergic rhinitis (or history of atopic disease in first-degree relatives of children under 4 years of age); (3) history of dry skin all over the body in recent years; (4) presence of flexor side eczema (eczema of the cheeks/forehead and extremities in children under 4 years of age); (5) onset before 2 years of age (for patients over 4 years of age). Atopic dermatitis was diagnosed when there was pruritus with three or more secondary criteria in infants and toddlers.

### Statistical analysis

Descriptive statistics were used to summarize the general demographic characteristics of all participants. Mean (standard deviation [SD]) was used for continuous variables and frequency (%) was implemented for categorical variables. Independent t-tests, Mann-Whitney U test, Chi-square test and Fisher exact tests were utilized to compare the relationship between variables, including demographics, environmental factors and diet during pregnancy and the subsequent risk for development of atopic dermatitis in infants and toddlers.

For the microbiota data, raw 16S rRNA sequencing data were spliced together, with quality control and filtering conducted by FLASH (https://ccb.jhu.edu/software/FLASH/index.shtml). The UPARSE version 7.0.1090 (http://drive5.com/uparse/) was used to cluster the smallest operational classification units (OTUs) based on highly similar sequences (>97%). Chimeric sequences were identified and removed using UCHIME in the process of clustering. The RDP classifier bayesian algorithm version 2.11 (http://sourceforge.net/projects/rdp-classifier/) was used on the QIIME (QIIME version 1.9.1) platform for classification analysis of representative sequences of OTUs from the sliva138/16S bacterial classification database with a default confidence threshold of 0.7. Core species analysis was used to indicate that the sample size was sufficient (27). The fecal alpha diversity among the two groups was evaluated *via* Sobs, Shannon and Simpson indices. Principal coordinates analysis (PCoA) based on Bray-Curtis distance matrix was used to evaluate the beta diversity at the OTU level, and analysis of similarities (ANOSIM) was used to compare differences in beta diversity between groups. Wilcoxon rank-sum test was used to analyze the difference between the two groups for phylum and genus. After adjusting confounders (mothers: maternal age, mother’s educational level, alcohol intake during pregnancy and frequency of maternal soy products consumption; infants and toddlers: mode of delivery, breastfeeding mode, maternal alcohol intake during breastfeeding, antibiotics exposure and pet exposure), logistics regression analysis was used to further explore the differential genera among atopic dermatitis and control groups.

The p-values for multiple analyses were adjusted using the Benjamini-Hochberg false discovery rate (FDR). The significant level and FDR threshold were both at 0.05. SPSS 23.0 (IBM, Chicago, IL, USA) and R 4.0.2. were used for all data analyses.

## Results

### General characteristics and factors affecting the development of atopic dermatitis

Characteristics and medical history of the mother and offspring pairs were displayed in **Table 1**. Mean age of the pregnant women was 29.83 (SD = 3.14) years. More than half of the pregnant women for both groups had a bachelor’s degree or higher. Infants of both groups were full-term with a mean weight of 3.40 (SD = 0.36) kg and a mean height of 50.41 (SD = 1.35) cm. The cesarean delivery rate was 61.1%, with a breastfeeding rate of 58.3% for the first 6 months postpartum. Comparatively, although there was no significant different of breastfeeding between control group and the atopic dermatitis group, the breastfeeding rate for the atopic dermatitis group was lower than that of the control group (30.8% vs. 69.2%). There were no statistical differences in baseline characteristics of the mother and offspring among atopic dermatitis and control groups. The results did not demonstrate any significant associations between environmental variables, diet during pregnancy, and subsequent risk of atopic dermatitis in infants and toddlers (**Table 1** and **Supplemental Table 1**).

**Table 1:** Demographic and environmental variables characteristics of the participants.

Characteristics		Atopic dermatitis		P value
	All	No (n=26)	Yes (n=10)	
**Demographic variables**				
Mother’s age (years)*	29.83 ± 3.14	29.42 ± 2.64	30.90 ± 4.15	0.211
Mother’s educational level				1.000
college degree or below	12 (33.3%)	8 (34.8%)	4 (40.0%)	
bachelor’s degree	20 (55.6%)	14 (60.9%)	6 (60.0%)	
master degree or above	1 (2.8%)	1 (4.3%)	0 (0%)	
Gestational age (weeks)*	39.4 ± 0.914	39.40 ± 0.87	39.40 ± 1.08	1.000
Gender of the infant				0.454
Female	25 (69.4%)	19 (73.1%)	6 (60.0%)	
Male	11 (30.6%)	7 (26.9%)	4 (40.0%)	
Birth Weight of the infant (kg)*	3.40 ± 0.36	3.38 ± 0.39	3.47 ± 0.27	0.481
Birth Height of the infant (cm)*	50.41 ± 1.35	50.50 ± 1.44	50.20 ± 1.13	0.565
The infant is the first child				0.673
Yes	25 (69.4%)	18 (78.3%)	7 (70.0%)	
No	8 (22.2%)	5 (21.7%)	3 (30.0%)	
**Environmental variables**				
Mode of delivery				0.462
Cesarian section	22 (61.1%)	17 (65.4%)	5 (50.0%)	
Vaginal	14 (38.9%)	9 (34.6%)	5 (50.0%)	
Breastfeeding in first 6 months				0.058
Yes	21 (58.3%)	18 (69.2%)	3 (30.0%)	
No	15 (41.7%)	8 (30.8%)	7 (70.0%)	
Alcohol intake during pregnancy				0.193
Yes	9 (25.0%)	5 (19.2%)	4 (40.0%)	
No	27 (75.0%)	21 (80.8%)	6 (60.0%)	
Alcohol intake during breastfeeding				0.658
Yes	8 (22.2%)	5 (19.2%)	3 (30.0%)	
No	28 (77.8%)	21 (80.8%)	7 (70.0%)	
Antibiotics using in the first 6 months				0.269
Yes	8 (22.2%)	7(26.9%)	1 (10.0%)	
No	28 (77.8%)	19 (73.1%)	9(90.0%)	
Antibiotics using in the first 1 years				0.092
Yes	19 (52.8%)	16 (61.5%)	3 (30.0%)	
No	17 (47.2%)	10 (38.5%)	7 (70.0%)	
Antibiotics using in the first 2 years				0.269
Yes	26 (72.2%)	20 (76.9%)	6 (60.0%)	
No	10 (27.8%)	6 (23.1%)	4 (40.0%)	

Data marked with * are presented as mean (standard deviation), all others are presented as frequency (%);

Continuous and categorical variables were compared between the two groups using independent t-test and chi-square test or Fisher’s exact test, respectively.

### Descriptions of the gut microbiota and changes of maternal and children’s gut microbiota

In total, 7,976,645 high-quality reads were obtained from the 169 stool samples. These reads were clustered into 2,673 OTUs. The flatness of the core species curve indicated that the sequencing sample size was sufficient (**Supplemental Figure 1**).

**Figure 1:**
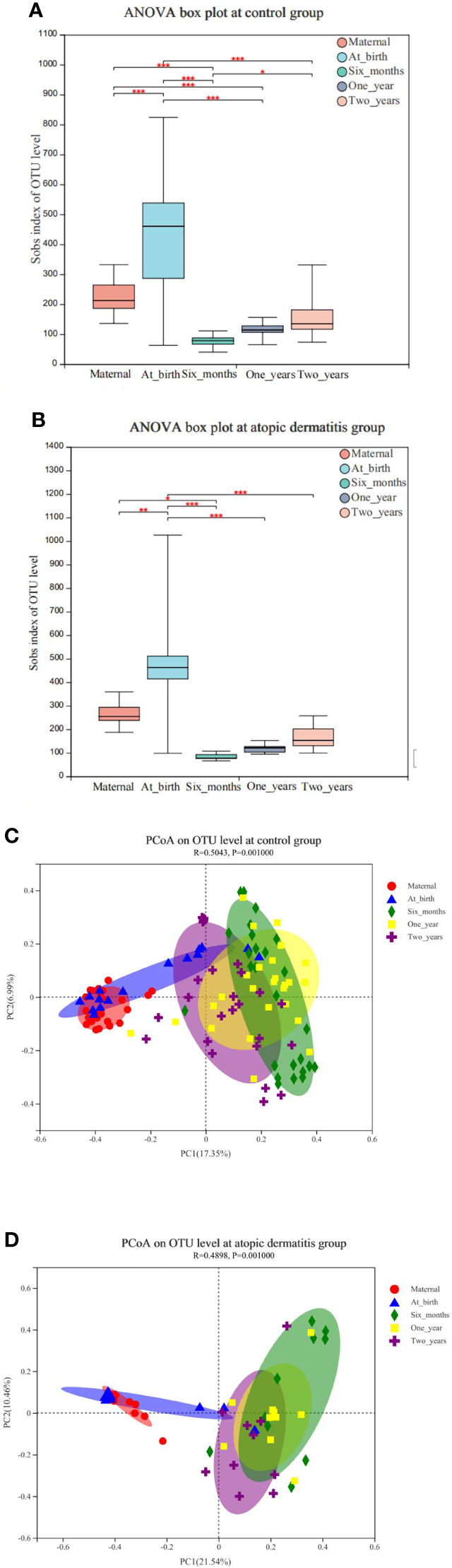
**(A)** Changes of maternal and infant and toddler’s microbial alpha diversity of in control group. **(B)** Changes of maternal and infant and toddler’s microbial alpha diversity in atopic dermatitis group. **(C)** Principal coordinate analysis (PCoA) analysis of the OTU level at maternal and infants and toddlers in control group. **(D)** Principal coordinate analysis (PCoA) analysis of the OTU level at maternal and infants and toddlers in atopic dermatitis group. **(A, B)** P value for alpha diversity differences at atopic dermatitis groups and control group was determined by one-way ANOVA (analysis of variance) after adjusting for subject. The 95% confidence interval around the mean is displayed by the boxplots. **(C, D)** The X-axis and Y-axis in Figure 1 represent the two selected principal axes, and the percentage represents the explanatory degree value of the principal axes to the sample composition difference; The scale of X axis and Y axis is relative distance and has no practical significance. Points with different colors or shapes represent samples of different groups. The more scattered the two sample points are, the greater the difference in species composition between the two samples. *0.01<P≤0.05, **0.001<P≤0.01, ***P≤0.001.

We conducted a within-group comparison of alpha diversity and beta diversity in infants and toddlers and their mothers in the atopic dermatitis groups and control group, respectively. The results showed that alpha diversity (Sobs, Shannon and Simpson indices) of infants and toddlers increased with age and the trends were similar in both groups (**Figure 1** and **Supplementary Figure 2**). It was found that in the control group, Sobs index was higher in infants at birth and lower at 6 months and 1 year in infants and toddlers compared to maternal Sobs index (**Figure 1A**). In addition, in the atopic dermatitis group, Sobs index was higher at birth and lower at 6 months compared to maternal Sobs index (**Figure 1B**). The results also revealed significant differences in beta diversity in the control group (**Figure 1C**) or in the atopic dermatitis group (**Figure 1D**) among different ages, and the maternal community composition was similar to the infants’ community composition at birth, while the maternal community composition was significantly different from that of infants and toddlers at 6 months, 1 year and 2 years (**Figure 1**).

In addition, we compared the differences in maternal alpha diversity and beta diversity between high or low frequency of different foods consumption (more than 3 days or 3 days/week vs less than 3 days/week). Our results didn’t find the difference in maternal alpha diversity (Shannon and Simpson indices) and beta diversity between high or low frequency of different foods consumption (**Supplemental Table 2** and **Supplemental Table 3**). However, our results found mothers who consumed soy products less than 3 days/week had higher Sobs index (**Supplemental Table 2**).

### Associations between maternal gut microbiota and atopic dermatitis of infants and toddlers

**Figure 2:**
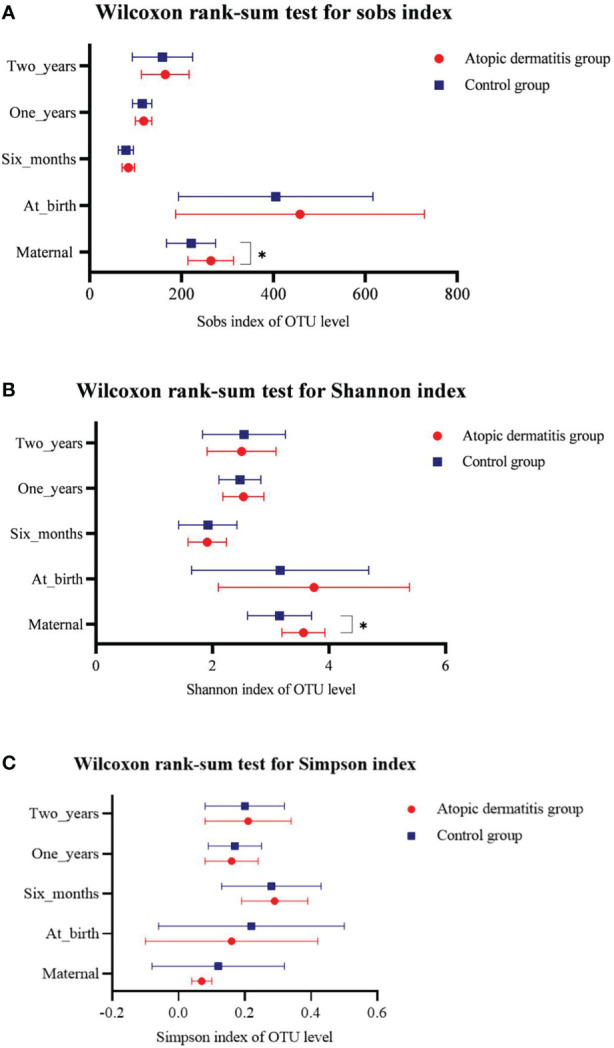
Differences of maternal and offspring’s microbial alpha diversity between Atopic dermatitis group and Control group. **(A)** Wilcoxon rank-sum text for sobs index. **(B)** Wilcoxon rank-sum text for Shannon index. **(C)** Wilcoxon rank-sum text for Simpson index.

**Figure 3:**
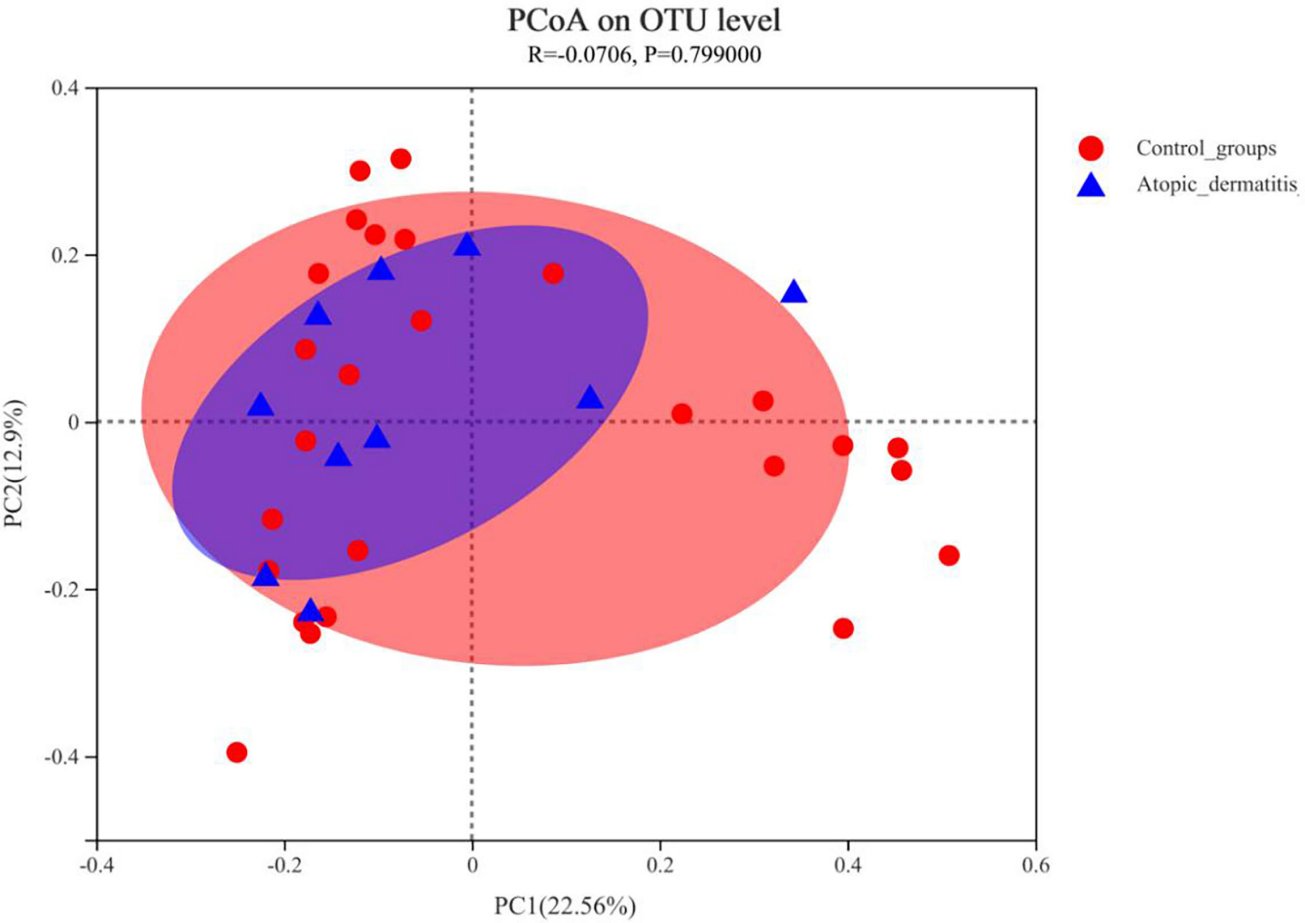
Principal coordinate analysis (PCoA) analysis of the OTU level at mother in atopic dermatitis group and control group. The X-axis and Y-axis in **Figure 1** represent the two selected principal axes, and the percentage represents the explanatory degree value of the principal axes to the sample composition difference; The scale of X axis and Y axis is relative distance and has no practical significance. Points with different colors or shapes represent samples of different groups. The more scattered the two sample points are, the greater the difference in species composition between the two samples.

We compared the diversity and composition of the gut microbiota perinatally in mothers of both groups. Surprisingly, this study indicated that the alpha diversity of maternal gut microbiota in control groups was higher than that of atopic dermatitis groups (Sob and Shannon indices all p < 0.05, **Figure 2**). It was noted that the Shannon index of maternal gut microbes between the atopic dermatitis group and control group was not statistically significant after controlling confounding factors (mother’s age, mother’s educational level, alcohol intake during pregnancy and frequency of maternal soy products consumption); however, the Sobs index still retains statistical significance. Based on the PCoA plot, there were no significant differences in the beta diversity of maternal gut microbiota between the atopic dermatitis group and control group (R=-0.0706, p=0.799, **Figure 3**).

Compositions of maternal gut microbiota at the phylum and genus level were displayed in **Supplemental Figure 3A** and **Supplemental Figure 3B**. The predominant phyla in the atopic dermatitis group and control group were Firmicutes (59.94% vs. 59.74%) and Bacteroidetes (35.10% vs. 35.97%), respectively. Twenty-two genera accounting for more than 0.01% were identified in each group with the top five genera as follows: *Bacteroides* (26.9% vs. 18.9%), *Faecalibacterium* (11.36% vs. 14.45%), *Prevotella* (3.75% vs. 13.73%), *Agathobacter* (4.49% vs. 4.51%) and *Phascolarctobacterium* (4.53% vs. 3.15%). Following the interpretation of the aforementioned results, we discovered that the abundance of *Bacteroides* was higher in the atopic dermatitis group, while the abundance of *Prevotella* was higher in the control group, although no statistical difference was found in the subsequent analysis.

**Figure 4:**
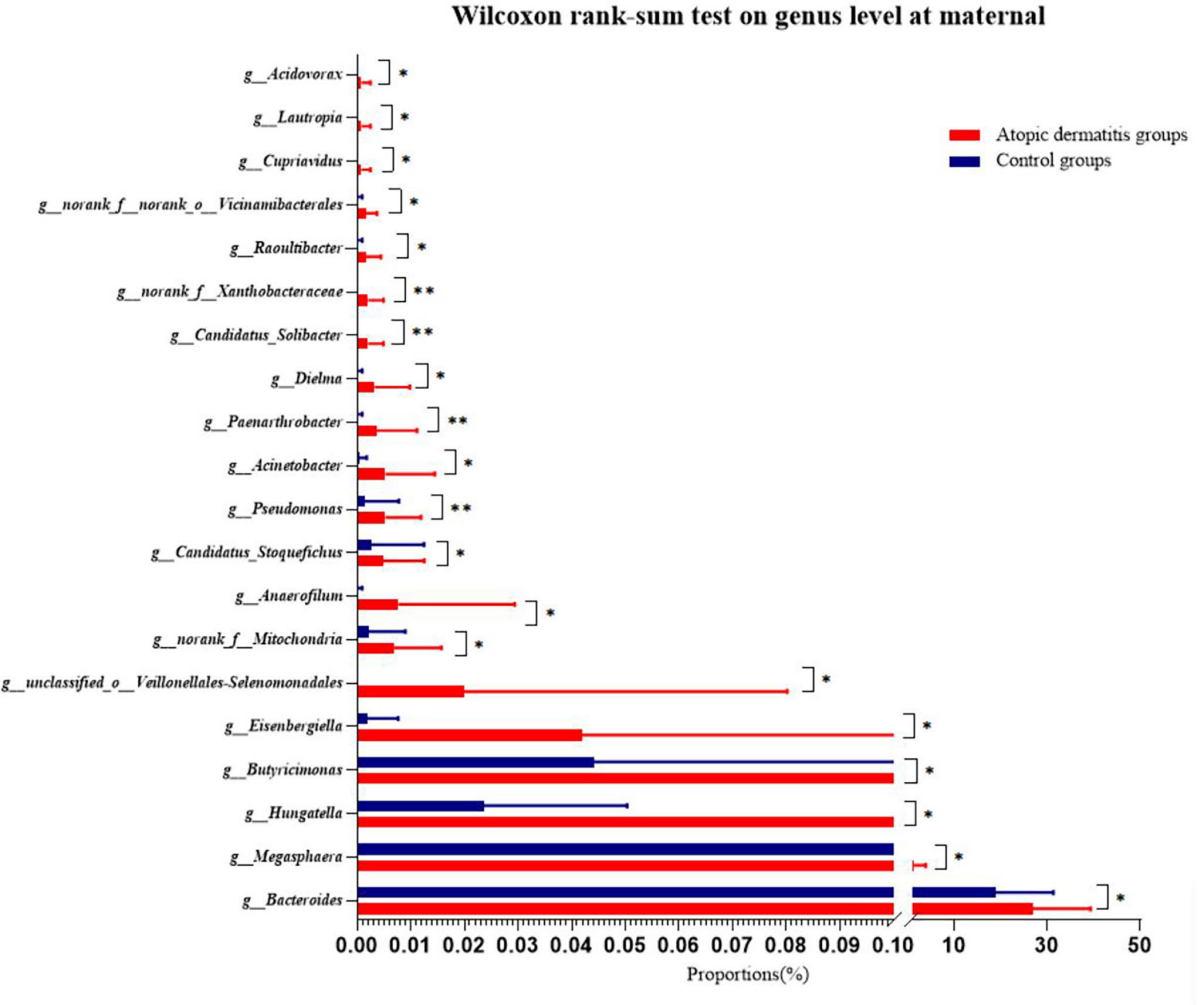
Differences of maternal gut microbiota of atopic dermatitis group and control group on genus level. Wilcoxon rank-sum test bar plot shows the differences of gut microbial composition between two groups on the genus level. The Y-axis represents different genera groupings, boxes of different colors represent two different groupings, and the X-axis represents the average relative abundance of genera in different groupings. * 0.01<P⩽0.05, **0.001 <P⩽0.01.

Wilcoxon rank-sum test was used to further investigate the differences in maternal gut microbiota composition between the atopic dermatitis group and the control group. The results found that at the phylum level, mothers in the atopic dermatitis group exhibited a lower abundance of *Fusobacteriota* compared to mothers in the control group, while *Acidobacteriota* was significantly higher in mothers of the atopic dermatitis group versus mothers in the control group (all p<0.05, **Supplemental Figure 3C**). In addition, maternal gut microbiota at the genus level in the atopic dermatitis group had higher abundance of *Bacteroides, Megasphaera, Hungatella, Butyricimonas, Eisenbergiella, Acinetobacter, norank_f_Xanthobacteraceae, Paenarthrobacter, unclassified_o_Veillonellales-Selenomonadales, Candidatus_Stoquefichus, norank_f_Mitochondria, Anaerofilum, Pseudomonas, Dielma, norank_f_Xanthobacteraceae* and *Candidatus_Solibacter* organisms than those in control group (**Figure 4**).

### Associations between children’s gut microbiota and atopic dermatitis of infants and toddlers

We performed group comparisons for infant and toddler microbial alpha diversity and beta diversity in both the atopic dermatitis group and the control group. This study did not demonstrate the difference in alpha diversity and beta diversity between the two groups in different ages (**Figure 2** and **Supplemental Figure 4**).

**Figure 5:**
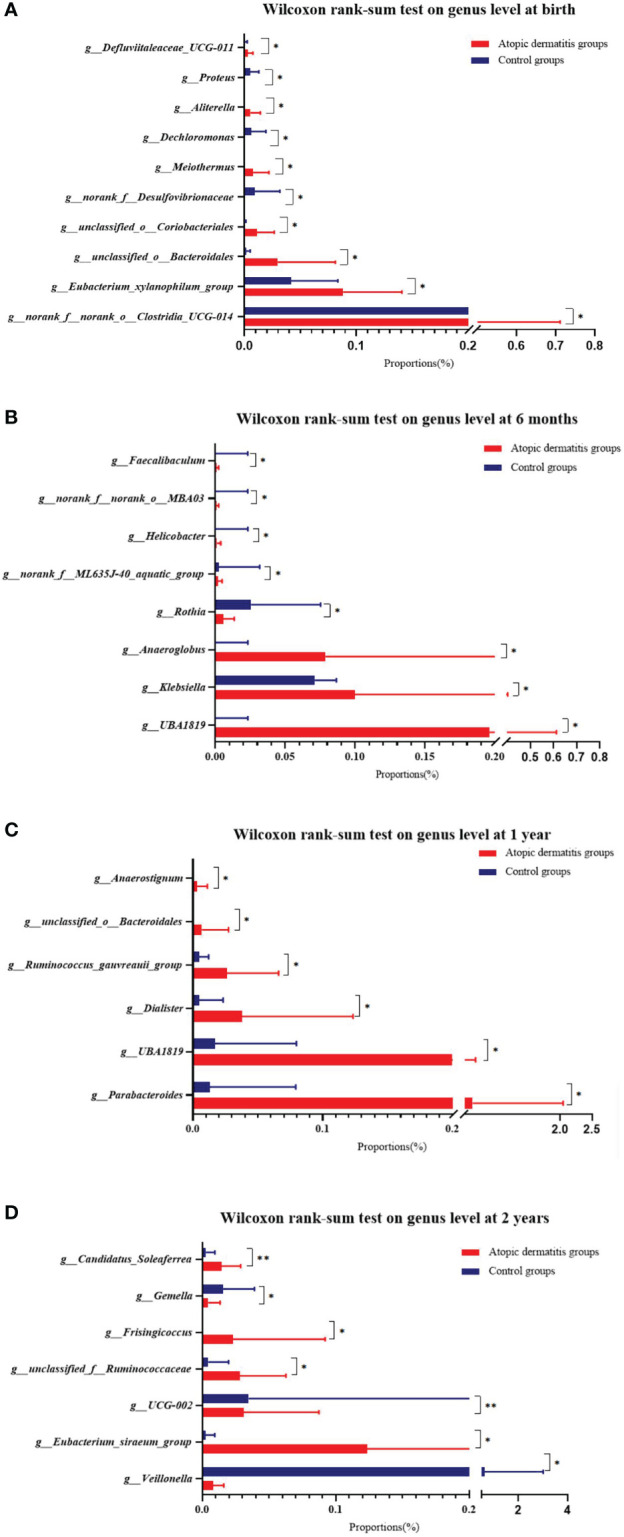
Difference of infant and toddler's gut microbiota of atopic dermatitis group and control group on genus level. **(A)** Wilcoxon rank-sum test on genus level at birth. **(B)** Wilcoxon rank-sum test on genus level at 6 months. **(C)** Wilcoxon rank-sum test on genus level at 1 year. **(D)** Wilcoxon rank-sum test on genus level at 2 years. Note: Wilcoxon rank-sum test bar plot shows the differences of gut microbial composition between two groups on the genus level. The Y-axis represents different genera groupings, boxes of different colors represent two different groupings, and the X-axis represents the average relative abundance of genera in different groupings. *0.01<P⩽0.05, **0.001<P⩽0.01.

The overall trend of change for each dominant gut microbiota in atopic dermatitis and control groups was similar from 0-2 years at the phylum level (**Supplemental Figure 5**). To further investigate the differences in gut microbiota composition between atopic dermatitis and control groups, we performed analyses based on the Wilcoxon rank-sum test. At the phylum level, the results revealed that the abundance of *Proteobacteria* was significantly lower in the atopic dermatitis group than in the control group of infants aged 1 year (p < 0.05). The results of the study revealed that the abundance of *norank_f_norank_o_Clostridia_UCG-014, unclassified_o_Coriobacteriales, Aliterella, Eubacterium_xylanophilum_group, Defluviitaleaceae_UCG-011, unclassified_o_Bacteroidales and Meiothermus* were significantly higher in the atopic dermatitis group than in the control group at birth (p < 0.05). The results of the study also revealed a higher abundance of *Klebsiella, Helicobacter, norank_f_ML635J-40_aquatic_group, Faecalibaculum, Anaeroglobus, UBA1819 and norank_f_norank_o_MBA03* in the atopic dermatitis group than the control group in infants aged 6 months (p < 0.05). The atopic dermatitis group of infants at 1 year demonstrated a significantly higher abundance of *Parabacteroides, Anaerostignum, UBA1819, unclassified_o_Bacteroidale, Dialister and Ruminococcus_gauvreauii_group* compared to the control group. Toddlers aged 2 years of the atopic dermatitis group demonstrated significantly higher levels of *Eubacterium_siraeum_group, Candidatus_Soleaferrea, unclassified_f_Ruminococcaceae and Frisingicoccus*, but exhibited lower levels of *Veillonella, UCG-002* and *Gemella* compared to the control group (p < 0.05). Other relevant results are detailed in **Figure 5**.

### Impacts of maternal and offspring’s gut microbiota on atopic dermatitis

To further clarify the effect of differential genera on the development of atopic dermatitis in infants and toddlers, we classified the above differential genera into high and low abundance models based on the median of the total sample. These findings were then included in the logistic regression model. After adjusting for relevant covariates (mothers: maternal age, mother’s educational level, alcohol intake during pregnancy and frequency of maternal soy products consumption; infants and toddlers: mode of delivery, breastfeeding mode, maternal alcohol intake during breastfeeding, antibiotics exposure and pet exposure), this study revealed that mothers in the atopic dermatitis group exhibited a higher abundance of *Pseudomonas and Candidatus_Stoquefichus.* Meanwhile, findings suggest that infants and toddlers in the atopic dermatitis group demonstrated a higher abundance of *Eubacterium_xylanophilum_group* at birth, *Ruminococcus_gauvreauii_group* at 1 year and *UCG-002* at 2 years, while exhibiting a lower abundance of *Gemella* and *Veillonella* at 2 years (**Table 2**).

**Table 2:** Logistics regression analysis to explore the impacts of maternal and offspring’s gut microbiota on atopic dermatitis after controlling for confounding factors.

	β	S.E.	Wald	P value
**Gut microbiota in Pregnancy women** ^a^				
*Candidatus_Stoquefichus*				
Low (ref)				
High	2.842	1.158	6.025	0.014
*Pseudomonas*				
Low (ref)				
High	3.127	1.124	7.736	0.005
**Gut microbiota in infants and toddlers**				
*Eubacterium_xylanophilum_group* (at birth) ^b^				
Low(ref)				
High	2.041	0.966	4.462	0.035
*Ruminococcus_gauvreauii_group* (one year) ^c^				
Low(ref)				
High	2.051	0.832	6.073	0.014
*Gemella* (two years) ^d^				
Low(ref)				
High	-2.644	1.211	4.762	0.029
UCG-002 (two years) ^d^				
Low(ref)				
High	3.006	1.453	4.278	0.039
*Veillonella* (two years) ^d^				
Low(ref)				
High	-3.175	1.351	5.522	0.019

Ref, Reference group of the categorical variable.

a Logistics regression analysis Model 1, adjusting covariates (mother’s age, mother’s educational level, alcohol intake during pregnancy and frequency of maternal soy products consumption).

b Logistics regression analysis Model 2; adjusting covariates (mode of delivery).

c Logistics regression analysis Model 3; adjusting covariates (mode of delivery, maternal alcohol intake during breastfeeding, breastfeeding in first 6 months, antibiotics using in the first 6 months and pet exposure).

d Logistics regression analysis Model 3; adjusting covariates (mode of delivery,maternal alcohol intake during breastfeeding, breastfeeding in first 6 months, antibiotics using in the first 6 months and pet exposure).

## Discussion

This was the first study to explore the effects of involving maternal-offspring microbiota status on the subsequent risk for development of atopic dermatitis in infants and toddlers up to two years. Our findings suggested that the enrichment and reduction of certain gut microbiota were strongly associated with the development of atopic dermatitis in infants and toddlers. These findings provide a basis for the development of interventions in the risk reduction and treatment of atopic dermatitis in infants and toddlers.

Our results indicated that there was a lower abundance of *Prevotella* in mothers assigned to the atopic dermatitis group versus the control group. But no statistical difference was noted in the subsequent analysis, likely due to the small sample size. However, these results reflected to some extent that the enrichment of *Prevotella* during pregnancy may serve as a protective factor against the development of pediatric allergic diseases. Vuillermin et al. (24) discovered that an increased abundance of *Prevotella* in pregnant women was associated with a decreased risk for the development of food allergies in respective offspring. *Prevotella* is a gram-negative anaerobic bacterium that ferments dietary fiber to produce metabolites, including short-chain fatty acids (SCFAs) and succinic acid (28, 29). SCFAs demonstrate significant anti-inflammatory effects and may influence fetal immune development through the production of interleukin-10 (IL-10) producing regulatory T cells (11). Additionally, succinic acid can stimulate the development, migration, and function of innate immune cells (30). In addition, *Prevotella* produces endotoxins that negatively affect fetal immune development and increase the risk for allergic outcomes *via* toll-like receptor-4-dependent pathways (31).

Previous work (32) indicated that an increased abundance of *Bacteroides*, an anaerobic bacterium belonging to the *Bacteroidaceae*, promoted the secretion of IL-6 and IL-23 in dendritic cells. IL-6 and IL-23 can promote the differentiation of Th17 cells and the secretion of IL-17. Th17 cells trigger inflammatory pathways that increase the risk for development of chronic autoimmune and allergic disease states (33). Therefore, the association between increased *Bacteroides* and the risk for atopic dermatitis is further understood by *Bacteroides*-associated cytokine production with notable systemic downstream effects. Our study further supports this finding in that a higher abundance of *Bacteroides* was noted in mothers of infants with atopic dermatitis, without adjusting for covariates. Our results also revealed maternal carrier with the higher abundance of *Pseudomonas* and *Candidatus Stoquefichus* during pregnancy was risk factor for atopic dermatitis in infants and toddlers after the adjustment of potential covariates. *Pseudomonas* belonging to the *Proteobacteria* phylum is an opportunistic pathogen, it has been shown to induce a type 2 immune response leading to the production of mucin, which is used as an energy source by pathogens (34, 35). *Candidatus Stoquefichus* belongs to the class of *Bacilli.* Notably, there was limited data regarding the biological implications of these genera in atopic dermatitis. Further investigation of these genera as it relates to human application involving the gut microbiota should be considered in future studies.

Initially, our findings indicated a higher alpha diversity of the gut microbiota in mothers of the atopic dermatitis group compared to mothers in the control group. However, the relationship between maternal Shannon index and infantile atopic dermatitis was not significant after controlling for relevant covariates, consistent with findings of previous work (36). Hiromi Tanabe et al. (36) reported an increase in the total diversity of maternal gut microbiota during pregnancy in the atopic dermatitis group compared to mothers in control group, however, this difference was not statistically significant. Although the relationship between maternal gut microbiota during pregnancy, and the risk for development of atopic dermatitis in respective offspring remains controversial, our results indicated that enrichment and reduction of certain maternal gut microbiota during pregnancy were associated with the subsequent development of atopic dermatitis in infants and toddlers. This data provided a new perspective for preventative and interventional measures to minimize the development of allergic diseases in infants and toddlers.

Our study included postpartum analysis involving the examination of offspring gut microbiota status up to two years of age, and the potential pathophysiological impact involving the microbiota-immune axis and risk for pediatric atopic dermatitis. Hong et al. (2) noted a higher abundance of *Klebsiella* in infants with documented atopic dermatitis (2). Rhoads et al. (37) reported a higher abundance of *Klebsiella* in infants with colic, further substantiating the potential systemic inflammatory effects of *Klebsiella*. A prior study (38) demonstrated an increased abundance of *Parabacteroides* in infants presenting with atopic dermatitis. Our study further supports this finding in that a higher abundance of *Parabacteroides* and *Klebsiella* was noted in infants with atopic dermatitis, without adjusting for covariates. Our results showed that infants and toddlers in the atopic dermatitis group presented with a higher abundance of *Eubacterium xylanophilum group* at birth*, Ruminococcus gauvreauii group* at 1 year *and UCG-002* at 2 years after adjusting for covariates. *Eubacterium xylanophilum group* and *Ruminococcus gauvreauii group* belong to the family of *Lachnospiraceae*. Previous study (39) noted a significant increase in the abundance of *Lachnospiraceae* in infants and toddlers presenting with atopic dermatitis. Additionally, Xu et al. (40) discovered that *Ruminococcus gauvreauii group* was positively associated with systemic immune response mechanisms mediated *via* pro-inflammatory cytokines including tumor necrosis factor-α (TNF-α), IL-1β, and IL-6. Therefore, *Ruminococcus gauvreauii group* can initiate an inflammatory signaling cascade that may facilitate the pathophysiological development of allergic disease processes, including pediatric atopic dermatitis. However, there was no direct association between the above genera and risk for the development of atopic dermatitis in infants and toddlers in previous study (16). Particularly, the role of the genus *UCG-002* in the development of atopic dermatitis in infants and toddlers was unclear. Further studies involving the above three genera would be needed to explore its role in mechanisms of allergic disease.

Our study demonstrated an important association involving reduced abundance of *Gemella* and *Veillonella* with an increased risk for atopic dermatitis in infants and toddlers. However, it is important to note that our findings were not entirely consistent with previous studies. Los-R et al. (41) discovered a positive correlation between the development of allergic disease in infants and presence of *Gemella*. Moreover, Huang et al. (42) noted a higher abundance of *Gemella* in infants presenting with asthma, while Simonyte et al. (43) indicated that reduced abundance of *Veillonella* in infancy was associated with an increased risk of asthma. Despite the notable discrepancies in previous literature findings, the anti-allergic effects of *Gemella* and *Veillonella* should be explored and considered in future applications*. Veillonella*, an anaerobic gram-negative coccus, can ferment lactic acid to propionate and acetate (44). *Gemella* is obligatory fermentative, producing either a mixture of acetic and lactic acids or an equimolar molar mixture of acetic acid and CO_2_ depending on the abundance of oxygen (45). Therefore, both *Gemella* and *Veillonella* can produce SCFAs. SCFAs, such as butyrate, propionate, and acetate, are primary energy sources with anti-inflammatory and immunomodulatory effects (46). Previous studies also found lower levels of fecal SCFAs (i.e., acetate, butyrate and valerate) were associated with the development of atopic dermatitis in infants (47, 48). These findings cannot be generalized at this time as they require further investigation in studies involving larger sample sizes.

Our findings couldn’t determine significant differences in the gut microbiota diversity between atopic dermatitis and control children involving infants and toddlers at different ages, inconsistent with other relevant studies (16, 21). We suspected that these inconsistencies may be a result of variable dietary habits of both maternal and infant origin. Differences in breastfeeding rates and maternal diet may affect the gut microbiota composition of infants and toddlers across different populations. Other plausible causes of discrepancy include individual environmental influences and underreported perinatal/postnatal factors. Therefore, a comprehensive analysis of all potential variables must be considered when exploring the complex relationship between gut microbiota and the development of pediatric atopic dermatitis.

While our findings did not indicate an association between exogenous influences and subsequent development of atopic dermatitis in infants and toddlers, prior studies have indicated otherwise (49, 50). A possible limitation influencing the overall results of our study includes our small sample size, which proved to be insufficient in detecting any statistical significance in exogenous influence and the development of pediatric atopic dermatitis. Therefore, it is imperative that we consider a larger sample size to control for these variables in an effort to elucidate the relationship among exogenous influences that may pose an indirect effect on infantile gut-microbiota status in the development of atopic dermatitis and other allergic processes.

Our study presents with strengths and weaknesses. Our study was a longitudinal cohort study allowing us to explore the relationship between gut microbiota in the first two years of life and risk for pediatric atopic dermatitis. Additionally, this served as a pilot study to investigate the relationship between changes in maternal gut microbiota during pregnancy and the development of atopic dermatitis in infants and toddlers, closing the gap in current research on maternal gut microbiota and subsequent atopic dermatitis of infants and toddlers in China. However, there were some distinct limitations of our study that require rectification for future applications. Principally, the sample size was insufficient for generalization of the data obtained. Moreover, we did not collect a complete medical history of both maternal and paternal subjects which is necessary for determining additional genetic components that may contribute to the development of pediatric atopic dermatitis. 16S rRNA sequencing was also a limitation of this study as it cannot provide sequences in a resolution like shotgun sequencing and provides no information about the functional capacity of the gut microbiota. Additionally, a further genetic function prediction or the measurements of SCFAs are suggested to be conducted to explore the clinical relevance to the changes in fecal microbiota in future studies.

Results of this study supported that enrichment and reduction of certain gut microbiota in mother-offspring pairs were associated with an increased risk of atopic dermatitis in infants and toddlers. The enrichment of *Gemella* and *Veillonella* in the microbiota of offspring appear to exhibit protective properties against the development of atopic dermatitis. Moreover, the results indicated that the enrichment of *Prevotella* during pregnancy may serve as a protective factor in the development of allergic diseases in offspring. The enrichment of *Pseudomonas*, *Candidatus Stoquefichus* during pregnancy and *Ruminococcus gauvreauii group, Eubacterium xylanophilum group* and *UCG-002* in offspring were risk factors for the development of atopic dermatitis in the offspring. Collectively, these findings provide a basis for continued research involving the gut-microbiota-immune axis and for the development of target-specific interventions in the prevention of pediatric atopic dermatitis.

## Data availability statement

The datasets presented in this study can be found in online repositories. The names of the repository/repositories and accession number(s) can be found below: https://www.ncbi.nlm.nih.gov/, PRJNA482931.

## Ethics statement

The studies involving human participants were reviewed and approved by the Research Ethics Boards of Medical School of Wuhan University (JKHL2017-03-03). Written informed consent to participate in this study was provided by the participants’ legal guardian/next of kin.

## Author contributions

XF wrote all sections of the manuscript and performed the analysis. XF and YL contributed to the conception and design of the study. TZ, JD, and NW contributed to data collection. CH, JB, and YL contributed to the manuscript revisions. All authors contributed to the article and approved the submitted version.

## Funding

This work was supported by the National Natural Science Foundation of China (grant number 81903334).

## Acknowledgments

We were grateful for the technical support from Shanghai Majorbio Bio-pharm Technology Co., Ltd.

## Conflict of interest

The authors declare that the research was conducted in the absence of any commercial or financial relationships that could be construed as a potential conflict of interest.

## Publisher’s note

All claims expressed in this article are solely those of the authors and do not necessarily represent those of their affiliated organizations, or those of the publisher, the editors and the reviewers. Any product that may be evaluated in this article, or claim that may be made by its manufacturer, is not guaranteed or endorsed by the publisher.
