# Supplementary figures and images for: The associations of maternal and children’s gut microbiota with the development of atopic dermatitis for children aged 2 years
Source: Front Immunol. 2022 Nov 17;13:1038876. doi: 10.3389/fimmu.2022.1038876 (PMC9714546; doi:10.3389/fimmu.2022.1038876)

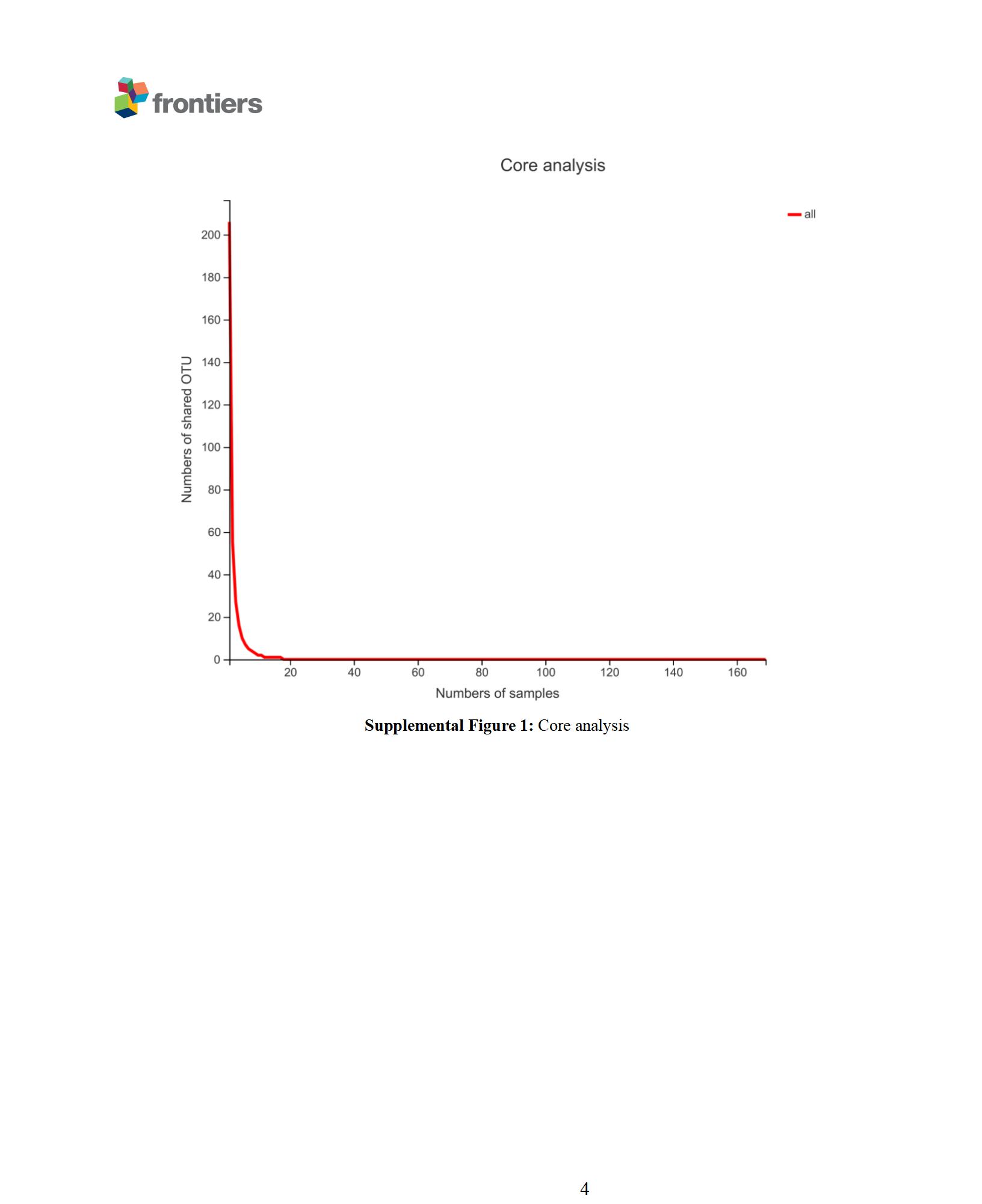

Supplement: Supplementary file 1 [file Image_1.jpeg]

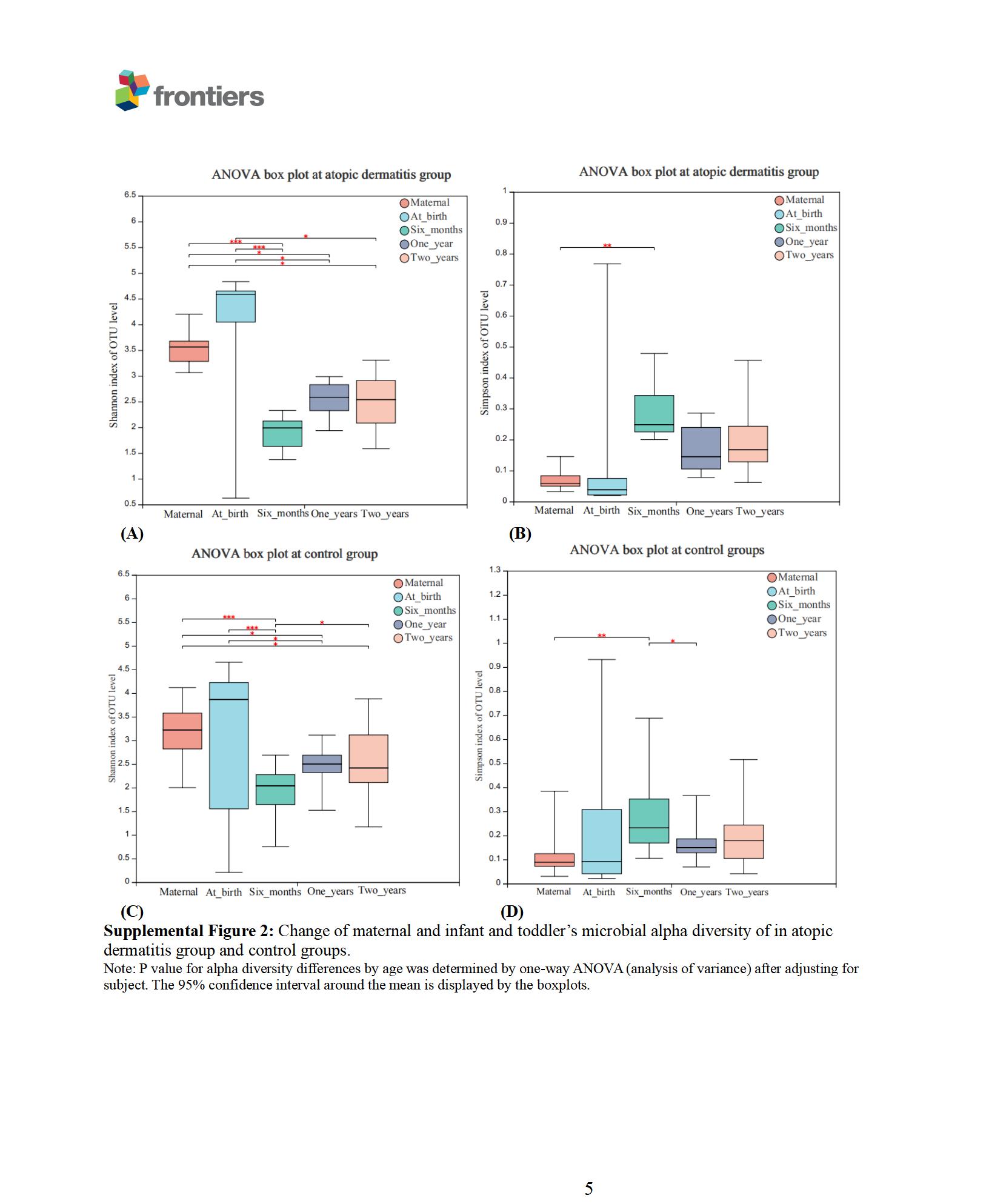

Supplement: Supplementary file 2 [file Image_2.jpeg]

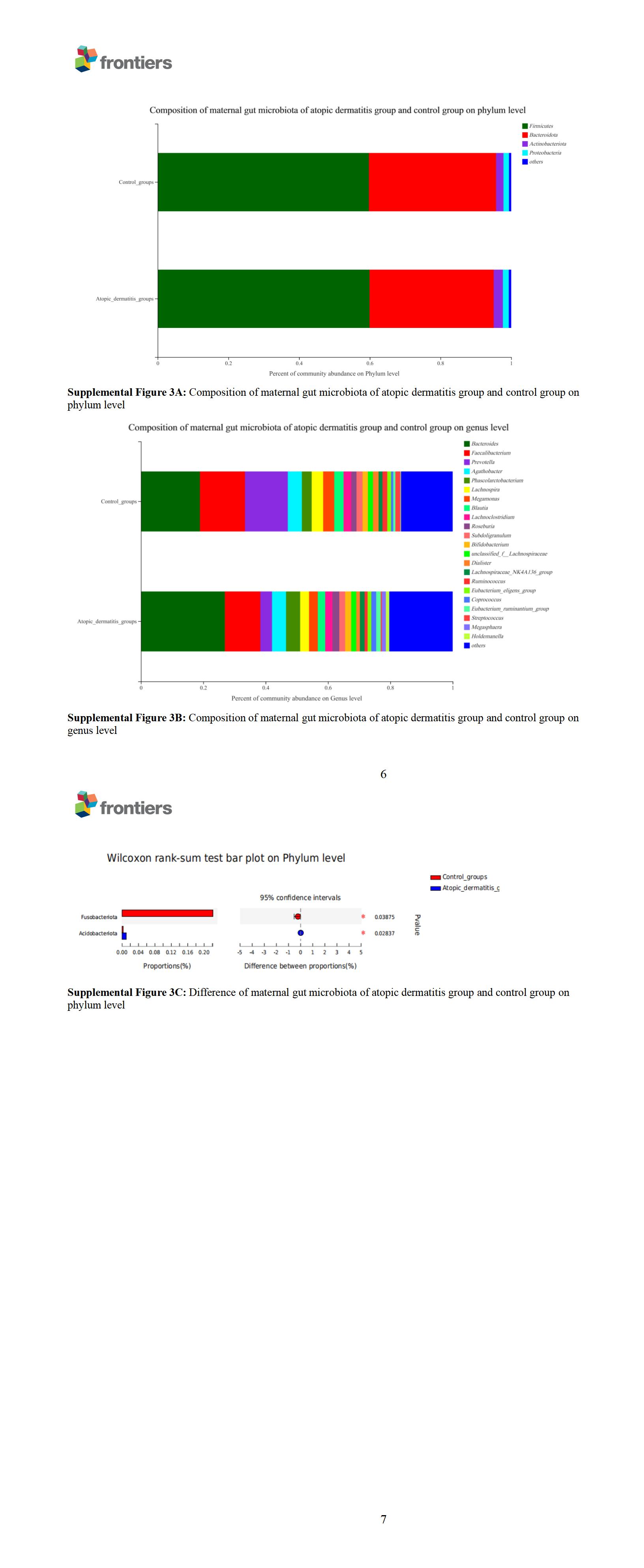

Supplement: Supplementary file 3 [file Image_3.jpeg]

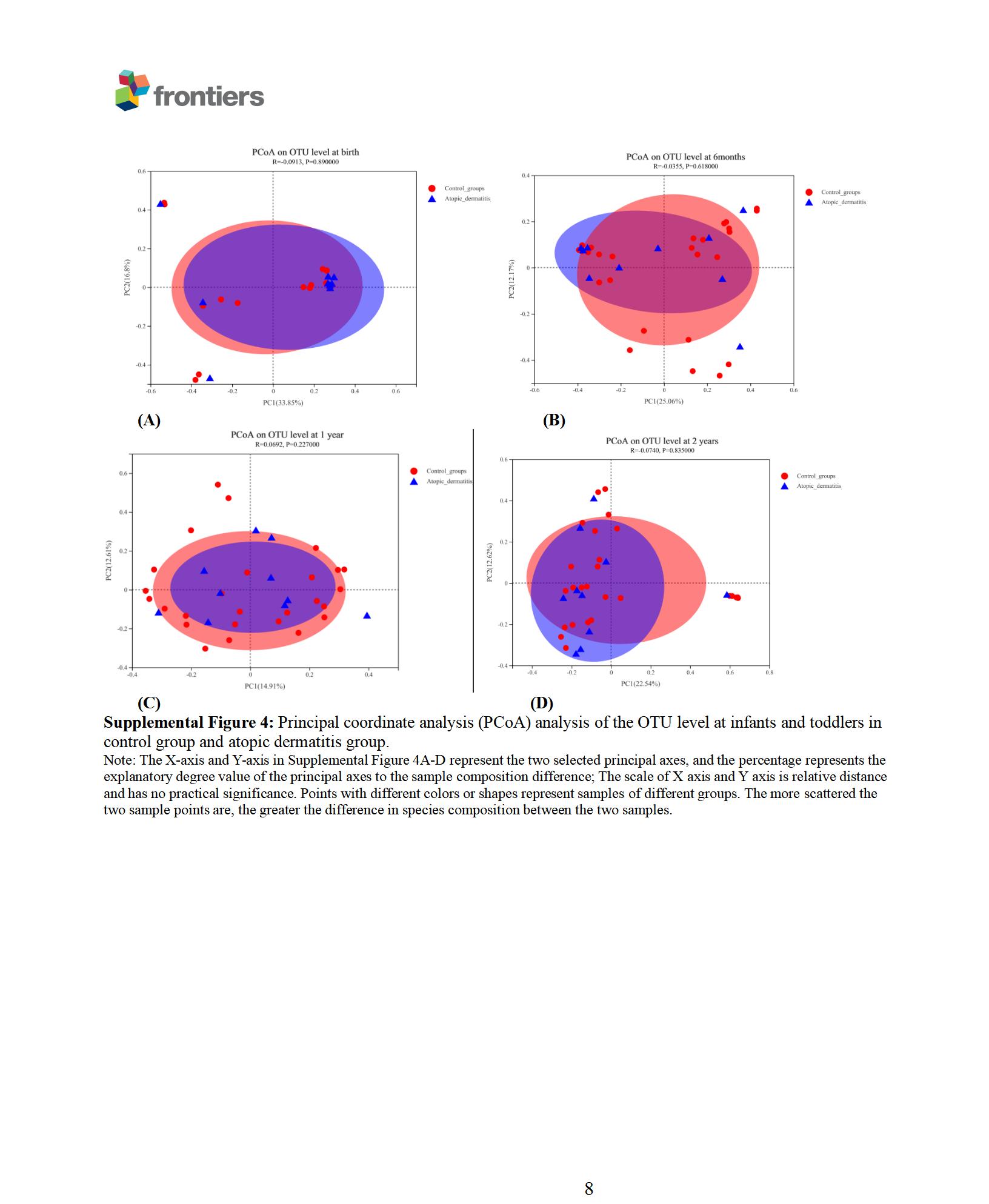

Supplement: Supplementary file 4 [file Image_4.jpeg]

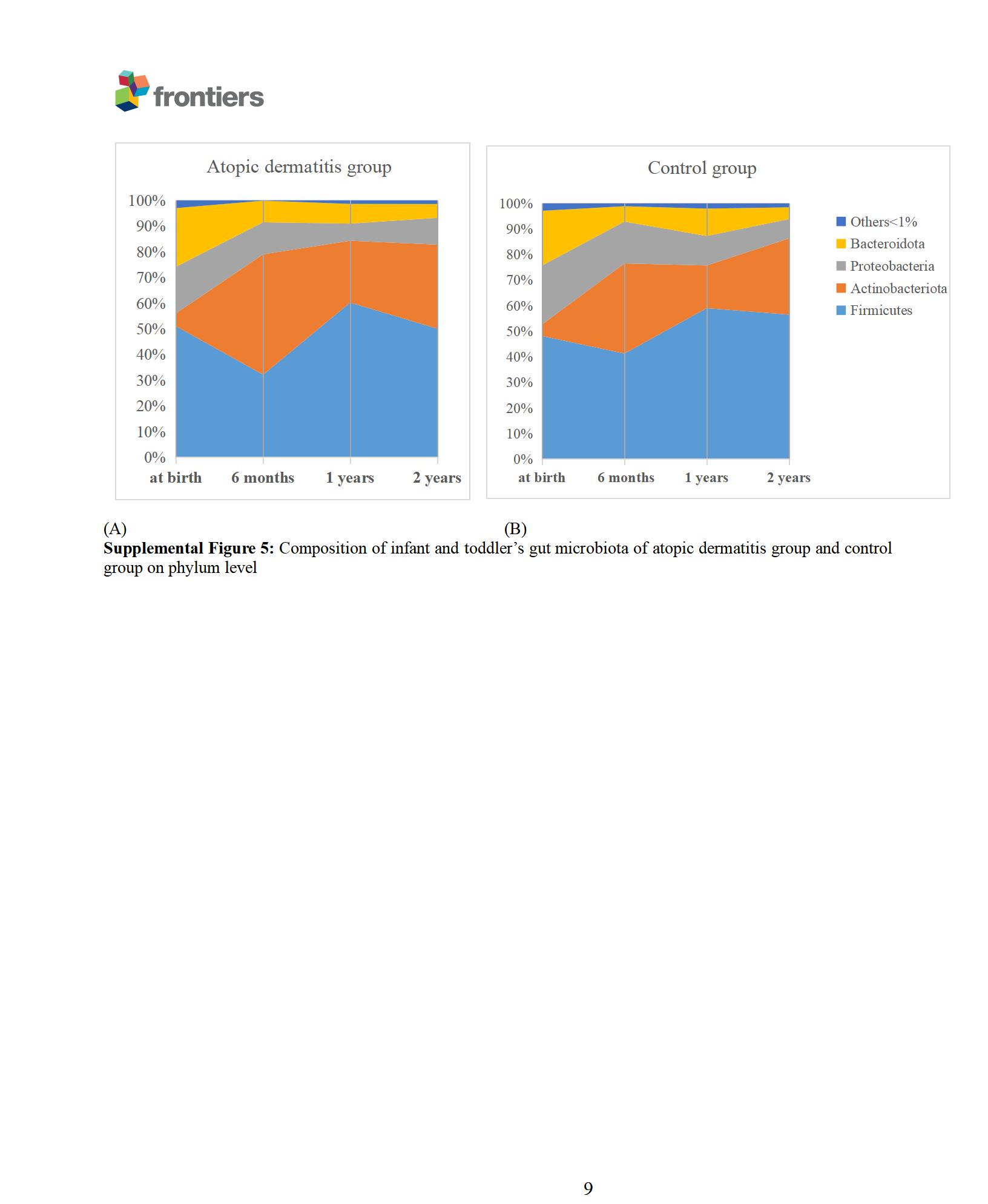

Supplement: Supplementary file 5 [file Image_5.jpeg]
