# Supplementary material for: The associations of maternal and children’s gut microbiota with the development of atopic dermatitis for children aged 2 years
Source: Front Immunol. 2022 Nov 17;13:1038876. doi: 10.3389/fimmu.2022.1038876 (PMC9714546; doi:10.3389/fimmu.2022.1038876)
Supplement: Supplementary file 6 [file Table_1.docx]

Supplementary Material

**Supplemental Table 1.** Diet frequency difference between atopic dermatitis group and control group(N=36)

| **Variables** | **Atopic dermatitis group**  **(n=10)** | **Control group**  **(n=26)** | **P value ^a^** |
| --- | --- | --- | --- |
| Frequency of meat consumption |  |  | 0. 119 |
| More than 3 days or 3 days/week | 7 (70.0%) | 24 (92.3%) |  |
| Less than 3 days/week | 3 (30.0%) | 2 (7.7%) |  |
| Frequency of dairy consumption |  |  | 0.688 |
| More than 3 days or 3 days/week | 8 (80.0%) | 17 (65.4%) |  |
| Less than 3 days/week | 2 (20.0%) | 9 (34.6%) |  |
| Frequency of vegetables consumption |  |  | 0.278 |
| More than 3 days or 3 days/week | 9 (90.0%) | 26 (100%) |  |
| Less than 3 days/week | 1 (10.0%) | 0 (0%) |  |
| Frequency of fruit consumption |  |  | 0.278 |
| More than 3 days or 3 days/week | 9 (90.0%) | 26 (100%) |  |
| Less than 3 days/week | 1 (10.0%) | 0 (0%) |  |
| Frequency of eggs consumption |  |  | 1.000 |
| More than 3 days or 3 days/week | 7 (70.0%) | 19 (73.1%) |  |
| Less than 3 days/week | 3 (30.0%) | 7 (26.9%) |  |
| Frequency of fish and shrimp consumption |  |  | 1.000 |
| More than 3 days or 3 days/week | 2 (20.0%) | 7 (26.9%) |  |
| Less than 3 days/week | 8 (80.0%) | 19 (73.1%) |  |
| Frequency of nuts consumption |  |  | 1.000 |
| More than 3 days or 3 days/week | 4 (40.0%) | 9 (34.6%) |  |
| Less than 3 days/week | 6 (60.0%) | 17 (65.4%) |  |
| Frequency of soy products consumption |  |  | 0.468 |
| More than 3 days or 3 days/week | 3 (30.0%) | 12 (46.2%) |  |
| Less than 3 days/week | 7 (70.0%) | 14 (53.8%) |  |

Note: ^a^ means using chi-square test or Fisher's exact method
