# Supplementary material for: The associations of maternal and children’s gut microbiota with the development of atopic dermatitis for children aged 2 years
Source: Front Immunol. 2022 Nov 17;13:1038876. doi: 10.3389/fimmu.2022.1038876 (PMC9714546; doi:10.3389/fimmu.2022.1038876)
Supplement: Supplementary file 7 [file Table_2.docx]

Supplementary Material

**Supplemental Table 2.** The association between diet during pregnancy and maternal alpha diversity

| **Characteristics** | More than 3 days or 3 days/week | Less than 3 days/week | P value^a^ |
| --- | --- | --- | --- |
| Frequency of meat consumption |  |  |  |
| Sobs index | 234.48±55.18 | 221.80±60.04 | 0.790 |
| Shannon index | 3.26±0.55 | 3.29±0.41 | 0.859 |
| Simpson index | 0.11±0.09 | 0.08±0.03 | 1.000 |
| Frequency of dairy consumption |  |  |  |
| Sobs index | 239.84±52.77 | 216.54±59.55 | 0.324 |
| Shannon index | 3.35±0.50 | 3.07±0.55 | 0.247 |
| Simpson index | 0.10±0.07 | 0.13±0.12 | 0.378 |
| Frequency of vegetables consumption |  |  |  |
| Sobs index | 232.40±55.92 | 244.000±0.00 | 0.833 |
| Shannon index | 3.26±0.54 | 3.47±0.00 | 0.778 |
| Simpson index | 0.11±0.09 | 0.06±0.00 | 0.667 |
| Frequency of fruit consumption |  |  |  |
| Sobs index | 232.40±55.92 | 244.00±0.00 | 0.833 |
| Shannon index | 3.26±0.54 | 3.47±0.00 | 0.778 |
| Simpson index | 0.11±0.08 | 0.06±0.00 | 0.667 |
| Frequency of eggs consumption |  |  |  |
| Sobs index | 236.69±58.37 | 222.40±46.90 | 0.639 |
| Shannon index | 3.22±0.59 | 3.38±0.33 | 0.590 |
| Simpson index | 0.12±0.10 | 0.07±0.02 | 0.303 |
| Frequency of fish and shrimp consumption |  |  |  |
| Sobs index | 236.444±60.86 | 231.481±54.30 | 0.641 |
| Shannon index | 3.19±0.46 | 3.29±0.56 | 0.651 |
| Simpson index | 0.12±0.07 | 0.10±0.09 | 0.330 |
| Frequency of nuts consumption |  |  |  |
| Sobs index | 230.92±48.95 | 233.74±59.40 | 0.948 |
| Shannon index | 3.21±0.46 | 3.30±0.57 | 0.580 |
| Simpson index | 0.11±0.09 | 0.10±.088 | 0.361 |
| Frequency of soy products consumption |  |  |  |
| Sobs index | 205.87±54.73 | 251.91±47.95 | 0.009 |
| Shannon index | 3.10±0.51 | 3.38±.52 | 0.077 |
| Simpson index | 0.11±.077 | 0.10±0.09 | 0.265 |

Note: ^a^ means using Wilcoxon rank-sum test.
