# Supplementary material for: The associations of maternal and children’s gut microbiota with the development of atopic dermatitis for children aged 2 years
Source: Front Immunol. 2022 Nov 17;13:1038876. doi: 10.3389/fimmu.2022.1038876 (PMC9714546; doi:10.3389/fimmu.2022.1038876)
Supplement: Supplementary file 8 [file Table_3.docx]

Supplementary Material

**Supplemental Table 3.** The association between diet during pregnancy and maternal beta diversity

| **Characteristics** | R^2^ | P value ^a^ |
| --- | --- | --- |
| Frequency of meat consumption | 0.031 | 0.352 |
| Frequency of dairy consumption | 0.017 | 0.923 |
| Frequency of vegetables consumption | 0.024 | 0.605 |
| Frequency of fruit consumption | 0.024 | 0.612 |
| Frequency of eggs consumption | 0.033 | 0.282 |
| Frequency of fish and shrimp consumption | 0.030 | 0.357 |
| Frequency of nuts consumption | 0.021 | 0.718 |
| Frequency of soy products consumption | 0.034 | 0.251 |

Note: ^a^ means using permutational multivariate analysis of variance.
